# Optimization of Cardiovascular Care: Beyond the Guidelines

**DOI:** 10.3390/jcm14072406

**Published:** 2025-04-01

**Authors:** Jakub Podolec, Tadeusz Przewłocki, Anna Kabłak-Ziembicka

**Affiliations:** 1 Department of Interventional Cardiology, Institute of Cardiology, Jagiellonian University Medical College, św. Anny 12, 31-007 Kraków, Poland; jjpodolec@gmail.com; 2 Department of Interventional Cardiology, The St. John Paul II Hospital, Prądnicka 80, 31-202 Kraków, Poland; tadeuszprzewlocki@op.pl; 3 Department of Cardiac and Vascular Diseases, Institute of Cardiology, Jagiellonian University Medical College, św. Anny 12, 31-007 Kraków, Poland; 4 Noninvasive Cardiovascular Laboratory, The St. John Paul II Hospital, Prądnicka 80, 31-202 Kraków, Poland

## 1. Introduction

Scientific cardiac and vascular societies provide essential guidelines tailored to broad patient populations. The most recent recommendations of the European Society of Cardiology (ESC), released in August 2024, focus on the management of arterial hypertension, chronic coronary syndromes, atrial fibrillation, and diseases of the aorta and peripheral arteries [1,2,3,4]. However, these guidelines lack a personalized approach, as they primarily address general cardiovascular risk factors, diagnostic strategies, and both pharmacological and non-pharmacological management. Consequently, some clinically significant but less mainstream conditions remain underrepresented or entirely absent in the guidelines. Personalized clinical strategies for specific patient subgroups are crucial, as they directly impact treatment adherence, quality of life, and long-term outcomes.

### 1.1. The Overlooked Aspects of Cardiovascular Care

ESC guidelines often fail to fully address the mental health aspects of cardiovascular disease (CVD), the role of polypharmacy and drug interactions, and the pathophysiological overlap between cardiovascular and non-cardiovascular disorders, including obstructive sleep apnea (OSA) and metabolic-associated steatotic liver disease (MASLD) [5,6,7,8].

### 1.2. Mental Health and Cardiovascular Disease

Mental disorders, including depression and frailty, significantly affect physical, psychological, and cognitive function in patients with cardiovascular disease, reducing the effectiveness of rehabilitation and adherence to medication regimens, that as a consequence considerably decreases life expectancy [5,9]. The prevalence of depression varies among CVD patients, averaging 20–30%, but rises to 41–51% in hospitalized patients with chronic coronary syndromes. Additionally, anxiety and depression levels remain elevated in up to 43% of patients during the first 12 months post-acute coronary syndrome [10,11]. Among patients with cardiac arrhythmia, depression prevalence is estimated at 24% in the general population, increasing to ca. 40% in older adults [12].

ESC guidelines recommend screening for depression using validated questionnaires, but detailed management strategies remain unspecified [1]. A collaborative guideline between psychiatric and cardiovascular societies could bridge this gap, offering clearer recommendations on the integration of mental health care into cardiology practice.

### 1.3. Obstructive Sleep Apnea and Cardiovascular Risk

Certain comorbidities share common pathophysiological pathways with atherosclerosis and cardiovascular risk factors [7,13,14]. Snoring, present in ca. 45% of men and 20% of women, serves as an early indicator of possible OSA. OSA is diagnosed in one-third of men and one-fifth of women who snore regularly. Chronic vascular inflammation and oxidative stress, triggered by intermittent hypoxia, contribute to the development of atherosclerosis, increasing the risk of coronary artery disease (CAD) and major adverse cardiovascular and cerebrovascular events (MACCE) [14,15]. Severe OSA (Apnea-Hypopnea Index ≥ 30) is a significant independent risk factor for stroke [7]. Given that OSA, CAD, and stroke share similar inflammatory pathways, OSA should be recognized as a key contributor to atherosclerosis and cardiovascular events [7,14,15].

### 1.4. Challenges in Managing Elderly Cardiovascular Patients

Guidelines often lack robust evidence-based approaches for patients aged ≥ 75–80 years, as elderly individuals have been systematically excluded from randomized controlled trials. With an aging population, this knowledge gap is increasingly problematic. Currently, 46.05 million Europeans (ca. 10.25%) are aged ≥ 75 years [16]. This group is particularly vulnerable to panvascular disease, multimorbidity, and high cardiovascular risk [17].

Although the new ESC guidelines address aortic and peripheral artery diseases, they fail to offer specific management strategies for elderly patients with multiple comorbidities, frailty, or dementia [18]. More than 20% of the European population has asymptomatic peripheral artery disease (PAD), 7–15% have asymptomatic carotid artery disease, and over 40% suffer from renovascular disease [17]. However, there is limited data on PAD prevalence among different ethnic groups, regional risk factor variations, and the adaptive mechanisms of vital organs such as the brain, kidneys, and intestines [19,20,21,22].

While ischemic preconditioning has gained interest as a method to enhance hypoxia tolerance in cerebrovascular patients, its application remains far less developed compared to supervised walking training in PAD [23,24]. Advanced imaging techniques, both non-invasive and invasive, can improve microvascular circulation assessment, tissue perfusion, and hemodynamic compromise detection [25,26,27,28]. Artificial intelligence (AI)-assisted imaging holds promise in optimizing vascular diagnostics, but further validation is needed.

### 1.5. Controversies in Polypharmacy and Primary Prevention

Despite the widespread presence of panvascular atherosclerosis, there is ongoing debate regarding polypharmacy in elderly patients. Notably, despite the high prevalence of hyperlipidemia, data on statin use for primary prevention in adults aged ≥ 70 years remain insufficient [29]. Current multicenter trials, including PREVENTABLE (NCT04262206) and STAREE (NCT02099123), aim to evaluate the impact of statins on MACCE and dementia in older adults [30].

In contrast, substantial evidence supports the cardiovascular benefits of newer glucose-lowering agents, including sodium-glucose cotransporter-2 inhibitors (SGLT2is), glucagon-like peptide-1 (GLP-1) receptor agonists, and dipeptidyl peptidase-4 (DPP-4) inhibitors [17,31,32]. Diabetes is a major risk factor for PAD in individuals aged ≥ 70 years [33,34]. In this population, these novel therapies offer multiple advantages, including cardioprotective and nephroprotective effects, reduced risk of hypoglycemia, and diuretic properties beneficial for heart failure and hypertension. The pleiotropic effects of GLP-1 receptor agonists and SGLT2is are of particular interest, particularly their antifibrotic properties and potential role in atherogenesis inhibition [31,33,35,36].

## 2. Conclusions

Future research should prioritize trials that address evidence gaps in elderly patients, diverse populations, mental health integration, cardiovascular and non-cardiovascular disease interactions, and advanced imaging techniques. The optimal incorporation of AI-driven diagnostics and the development of multidisciplinary standardized protocols will be crucial in advancing personalized cardiovascular care.
